# EA at PC6 Promotes Gastric Motility: Role of Brainstem Vagovagal Neurocircuits

**DOI:** 10.1155/2019/7457485

**Published:** 2019-07-15

**Authors:** Mengjiang Lu, Chienchih Chen, Wen Li, Zhi Yu, Bin Xu

**Affiliations:** Key Laboratory of Acupuncture and Medicine Research of Ministry of Education, Nanjing University of Chinese Medicine, Nanjing 210023, Jiangsu Province, China

## Abstract

**Background:**

We aimed to assess whether electroacupuncture (EA) at PC6 affects gastric motility via the vagovagal reflex and if so whether brainstem vagovagal neurocircuits and related transmitters are involved.

**Methods:**

Gastric motility was measured in male Sprague-Dawley (SD) rats by placing a small manometric balloon in the gastric antrum. The rats were subjected to control, sham surgery, vagotomy, sympathectomy, and microinjection group, including artificial cerebrospinal fluid, gamma-aminobutyric acid (GABA), and glutamic acid (L-Glu). The effect of EA at PC6 on gastric motility was measured. Moreover, electrophysiological testing was used to measure the effect of EA at PC6 on the parasympathetic and sympathetic nerves. In addition, artificial cerebrospinal fluid, L-Glu, and GABA have been microinjected into the dorsal motor nucleus of the vagus (DMV) to measure the changes in gastric motility and parasympathetic nerve discharge induced by EA at PC6.

**Key Results:**

EA facilitated the gastric motility in control group. In the vagotomy group, gastric motility was not affected by EA at PC6. However, in the sympathectomy group, gastric motility was similar to control group. Acupuncture at PC6 increased parasympathetic nerve discharge but not sympathetic nerve discharge. Furthermore, the microinjection of L-Glu into the DMV increased gastric motility, although EA at PC6 showed no remarkable change in this group. The injection of GABA reduced gastric motility and parasympathetic nerve discharge, but EA at PC6 significantly increased gastric motility and the parasympathetic nerve discharge in this group.

**Conclusions and Inferences:**

EA at PC6—primarily by inhibiting GABA transmission to DMV—reduced the inhibition of efferent vagal motor fibers and thus promoted efferent vagus nerve activity and increased gastric motility.

## 1. Introduction

The digestive system (DS) consists of the upper digestive tract and the lower digestive tract. The upper digestive tract is mainly regulated by the vagus nervous system, especially the vagovagal reflex [1]. The brainstem vagovagal neurocircuits mainly include the nucleus of the solitary tract (NTS) and the motor vagus dorsal nucleus (DMV), and the gastric motility is regulated by the efferent vagus nerve originating from neurons located in the DMV. Evidence suggests that the vagus pathways for increasing or decreasing gastric motility appear to be localized in different regions of the DMV. For example, the nonadrenergic, noncholinergic (NANC)-pathway neurons which inhibit gastric motility appear to be located in the caudomedial and rostrolateral divisions of the DMV, whereas the gastroexcitatory neurons are located in the more rostral and medial divisions of the DMV [2, 3]. Also, GABA and Glu are two common neurotransmitters in vagovagal neurocircuits which excite or inhibit gastric motility [4, 5]. And there are conspicuous differences of topographical distribution between glutamate uptake and GABA uptake [6].

Traditional body surface-stimulating therapies such as acupuncture and moxibustion are widely used to treat gastrointestinal disorders [7, 8]. Previous animal studies found that acupuncture on the limbs can activate the parasympathetic nerve and increase gastric motility [9, 10]. In clinical, PC6 was widely used in treated gastric disorder. The randomized controlled trial shows that EA at PC6 promotes gastric emptying and alleviates the symptom of dyspepsia [11]. In animal studies, EA at PC6 may reverse the Tacrine side effect which significantly increases the gastric distention-induced transient lower esophageal sphincter relaxations (TLESRs) [12]. Moreover, electric acupoint stimulation at PC6 significantly inhibits the frequency of transient lower esophageal sphincter relaxations (TLESR) and the rate of common cavity during TLESR in cats. Furthermore, C-Fos immunoreactivity and NOS reactivity in the solitarius (NTS) and dorsal motor nucleus of the vagus (DMV) were significantly decreased by EA at PC6 [13].

In the study, we hypothesized EA at Neiguan (PC6) influences gastric motility via brainstem vagovagal neurocircuits. To test this hypothesis, we measured intragastric pressure in vagus nerve and splanchnic nerve excision model to observe the role of autonomic nerve pathway of EA at PC6. We also measured the activity of the autonomic nerve using electrophysiology. Finally, we studied the brainstem vagovagal neurocircuits by microinjecting GABA, L-Glu into DMV; then, we investigated the intragastric pressure and vagus nerve activity.

## 2. Materials and Methods

### 2.1. Animals

Sprague-Dawley rats (male, 250–300 g; Model Animal Research Center of Nanjing Medical University, China) were used in this study. All animals were housed under controlled environmental conditions (22°C, 40–60% relative humidity, 12/12 h light/dark cycle) and were given free access to water and food. All animals were allowed 2 weeks of feeding adaptation. All experimental manipulations were undertaken in accordance with the Principles of Laboratory Animal Care and the Guide for the Care and Use of Laboratory Animals, published by the National Science Council, China.

### 2.2. Drugs

Among the drugs used in the experiments, urethane (U2500; Sigma, St. Louis, MO, USA) was intraperitoneally injected to anesthetized rats, and L-Glu (G1251-100G; Sigma), GABA (A2129-10G; Sigma), and artificial cerebrospinal fluid (R22153; Yuan Ye Biological Co., Ltd., Shanghai, China) were administered via microinjection prior to surgery, whereas penicillin (2011; Shandong Shengwang Pharmaceutical Co., Ltd., Shandong, China) was administered after the surgery.

### 2.3. Gastric Motility Recording

All rats in the study underwent fasting for 12 h, but were given free access to water. A small incision (approximately 1 cm) was made under the xiphoid process. A small balloon (approximately 2 mm in diameter) made of flexible rubber was inserted into the duodenum and placed in the gastric antrum. The balloon was filled with warm water (0.05–0.1 ml), to keep the pressure at about 0.1KPa H_2_O.

Pressure in the balloon was measured by a transducer (YP201; Chengdu Instrument Factory, Chengdu) through a polyethylene tube and then transmitted into a multipurpose polygraph (RM6240; Chengdu Instrument Factory). The signal was captured by RM6240 software. An electric heating board was used to maintain the animals' body temperature at 37°C ± 0.5°C during the test. At the end of experiments, the animals were killed with an overdose of urethane.

### 2.4. Parasympathetic and Sympathetic Nerve Discharge

To examine nerve discharge, rats were anesthetized with 8 mL/kg of 20% urethane intraperitoneally, an incision was made in the midline of the abdomen, and the posterior branch of gastric vagus nerve or the left splanchnic nerve was identified and isolated. The positive electrode was kept in contact with the nerve, and the negative electrode was kept in contact with the surrounding tissue. The liquid paraffin wax, preheated to 37°C, was used to cover the nerve to protect it. Nerve discharges were recorded using a preamplifier (NL100; CED, UK) and Micro1401-3 Bioelectric Module (NL125NL126; CED, UK), both of which were connected to biological signal acquisition and analysis system (Microl1401-3; CED, UK). Spike2 software was used to analyze the nerve discharge data. Discharge during acupuncture was compared to that before the treatment. A difference of >5% in these values was considered as an increase in nerve discharge. Equation (1) was used to indicate the change in percentage discharge.

(1) $$\begin{array}{c} Percentage\;\;change=\frac{Acup-preAcup}{preAcup}\times 100\%. \end{array}$$

### 2.5. Surgery

For vagotomy, rats were anesthetized with 8 mL/kg of 20% urethane intraperitoneally, an incision was made in the midline of the abdomen, and parasympathetic nerves on both sides were dissected. For sympathectomy, rats were anesthetized with 8 mL/kg of 20% urethane intraperitoneally, an incision was made in the midline of the abdomen, and the left greater splanchnic nerve was dissected. The animals in the sham control group underwent the same surgical procedure, but without nerve dissection. After surgery, the animals received penicillin (0.2 mL/d of 800 IU penicillin in 5 mL saline per rat, intramuscular) and were allowed 3 days of postoperative recovery.

### 2.6. Microinjection of the Dorsal Motor Nucleus of the Vagus

The animals were placed on a stereotaxic apparatus (Kopf Instruments, US) and kept in the prone position. The location of the dorsal nucleus of the vagus nerve was determined using Paxinos and Watson rat brain mapping (AP 13.8 mm, RL 0.5 mm, H 7.8 mm) [14]. The microinjection guide cannula (RWD Life Science, China) was anchored with dental cement. After the operation, the animals received penicillin (0.2 mL/d per rat, intramuscular) and were allowed 3 days to recover. Thereafter, the rats received artificial cerebrospinal fluid (0.1 mol/L, 0.2 *μ*L), L-Glu (0.1 mol/L, 0.2 *μ*L), and GABA (0.1 mol/L, 0.2 *μ*L) via microinjection syringe and pump which connected to cannula [15]. The drugs were administered at 0.2 *μ*l per minute for 1 minutes. The rats were divided into 3 groups depending on the drug they received.

At the end of the experiment, the rats were microinjected with pontamine sky blue (0.2 *μ*L), whereby the location of the dorsal nucleus of the vagus nerve could be determined. The rats were sacrificed via an anesthetic overdose, and they were decapitated immediately. The brains were removed and soaked in paraformaldehyde solution for slicing. The brain tissue was sectioned and observed under a light microscope to check for the blue marker. The location of the marker was identified by referring The Rat Brain in Stereotaxic Coordinates (Paxinos and Watson), which helped enhance the accuracy of nuclear localization (Figure 4(c)).

### 2.7. EA Stimulation

PC6 (Neiguan) is located between the palmar tendon and flexor carpi ulnaris. A pair of stainless-steel acupuncture needles (diameter, 0.3 mm) were inserted to a depth of 3 mm into the muscle layer at the left PC6. The needles were connected to Han's EA instrument (LH402A; Beijing Huawei Co., Ltd., China). The stimulating intensity was 2 mA, the frequency was 2/15 Hz, and stimulation time was 2 min.

Since EA interferes with nerve discharge, hand stimulation was used for the nerve discharge experiments. The frequency of stimulation was 2–3 Hz, and the stimulation time was 2 min.

### 2.8. Assessment of Gastric Motility

The intragastric pressure during EA was compared with that before needle insertion. If the percentage difference in the intragastric pressure before and after needle insertion was >5%, gastric motility was considered to have increased. Similarly, if the percentage difference in intragastric pressure before and after drug microinjection into the dorsal nucleus of the vagus nerve was >5%, gastric motility was considered to have increased. Equations (2) and (3) were used to indicate the percentage change in intragastric pressure.

(2) Percentage  change=EA−preEApreEA×100%

(3) Percentage  chage=Drug−preDrugpreDrug×100%

### 2.9. Experimental Procedure

In the first experiment, we recorded the gastric pressure to describe the characteristics of gastric motility during EA. The rats were divided into two groups (parasympathetic nerve discharge and sympathetic nerve discharge), and the nerve discharges were measured during EA.

In the second experiment, the rats were divided into four groups (control, sham control, sympathectomy, and vagotomy groups). We first cut the gastric vagal nerves of the rats and recorded gastric pressure during EA to evaluate the role of the parasympathetic pathway. Then, we cut the splenic greater splanchnic nerve and recorded gastric pressure during EA to evaluate the role of the sympathetic pathway in different rats.

In the last experiment, we divided animals into three groups (CSF group, L-Glu group, and GABA group) and microinjected CSF, L-Glu, and GABA into DMV. Then we measured the gastric pressure and parasympathetic discharge used EA PC6 to explore the mechanism of DMV pathway. There were 8 animals per group in all experiments.

### 2.10. Statistical Analysis

Data were analyzed using SPSS 23.0 (SPSS, Chicago, IL, USA) and GraphPad Prism 6.0 (GraphPad Software, La Jolla, CA, USA). Any 2 groups were compared using the independent sample* t*-test, and three groups were compared using ANOVA. All data are expressed as mean ± standard deviation. P < 0.05 indicates significance.

## 3. Results

### 3.1. Baseline Recording of Gastric Motility

The gastric motility was measured by intragastric pressure in anesthetized rats. The pressure was maintained at approximately 0.1kPa as baseline by expanding the volume of the balloon with warm water, and rhythmic contractions were recorded at a rate of 4–6/min with 0.2–0.3 kPa in amplitude.

### 3.2. Effect of EA at PC6 on Gastric Motility

**Figure 1:**
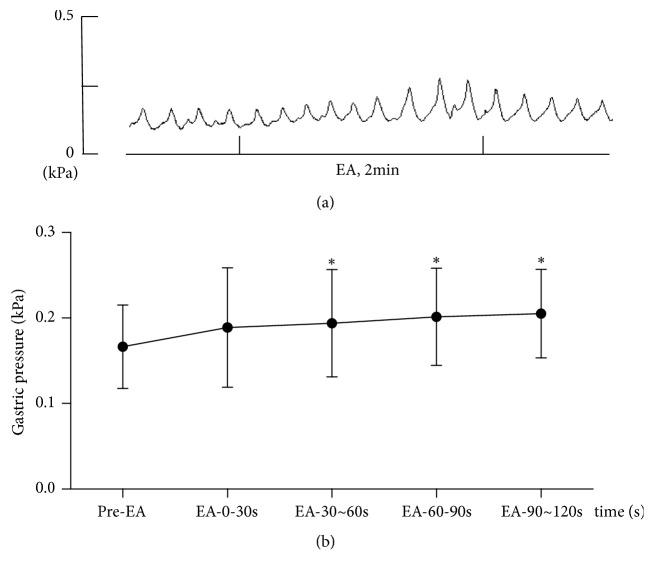
Effect of EA at PC6 on gastric motility. (a) Wave patterns of gastric motility changes in Sprague-Dawley rats that received EA stimulation at PC6. (b) Changes in the intragastric pressure in Sprague-Dawley rats that received EA stimulation at PC6 at different time intervals of stimulation (60 s before EA, and after EA for 30 s, 30–60 s, 60–90 s, and 90–120 s). ^*∗*^P < 0.05, compared with pre-EA (n = 8).

To investigate the effect of EA at PC6 on gastric motility, the intragastric pressure was recorded at different time points. The results showed that EA at PC6 can increase gastric motility; in fact, after 30 s of EA stimulation at PC6, the gastric motility was significantly different as compared to that before needle insertion (^*∗*^P < 0.05, n = 8; Figure 1).

### 3.3. Effect of EA at PC6 on Autonomic Nerve Discharge

**Figure 2:**
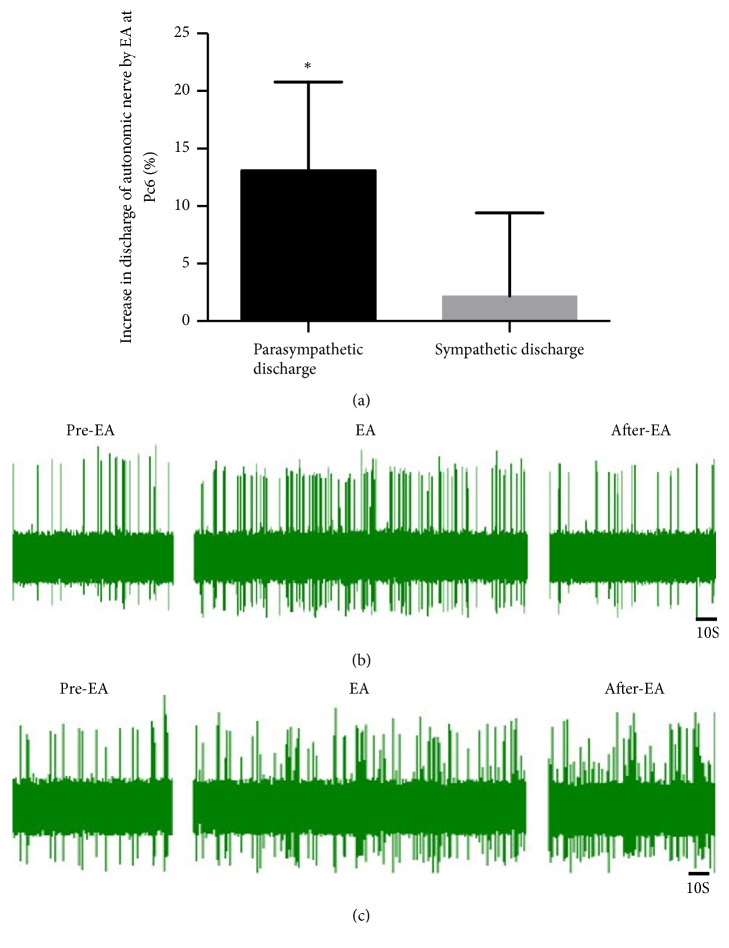
Effect of EA at PC6 on autonomic nerve discharge. (a) Percentage increase of parasympathetic nerve discharge and sympathetic discharge during EA. ^*∗*^P < 0.05, compared with sympathetic discharge (n = 8). (b) Parasympathetic waveform of EA at PC6. (c) Sympathetic waveform of EA at PC6.

The discharge from the parasympathetic and sympathetic nerves before and after EA was examined to assess the effects of acupuncture on the autonomic nerves. The results showed that parasympathetic nerve discharge after EA at PC6 was significantly higher than that before needle insertion, although there was no significant increase in splanchnic nerve discharge (n = 8; Figures 2(b) and 2(c)),). Moreover, the percentage increase in parasympathetic nerve discharge was significantly higher than the percentage increase in sympathetic nerve discharge (^*∗*^P < 0.05, n = 8; Figure 2(a)).

### 3.4. Effect of EA at PC6 on Gastric Motility after Sympathectomy and Vagotomy

To investigate whether EA at PC6 affects gastric motility via the sympathetic pathway or the parasympathetic pathway, the left splanchnic nerve and the parasympathetic nerves on both sides were severed in the rats. The results showed a significant increase in gastric motility in the control, sham control, and sympathectomy groups during EA, but no significant increase in gastric motility in the vagotomy group. The percentage increase in gastric motility via EA decreased significantly in the vagotomy group and differed significantly between the control and sham groups (P < 0.05). However, there are no differences among the control, sham control, and the sympathectomy groups (P>0.05). Furthermore, the baseline intragastric pressure reduced significantly after vagotomy. These results indicate that the parasympathetic pathway but not the sympathetic pathway plays an important role in the effect of EA at PC6.

### 3.5. Effect of L-Glu and GABA Microinjection into DMV on Gastric Motility

To further study the relationship between the central nervous system and the vagal nerve pathways, L-Glu and GABA were microinjected into the DMV, and the consequent changes in gastric motility were analyzed. The results showed that the gastric motility increased after L-Glu microinjection, but decreased after GABA microinjection (n = 8; Figure 4).

To confirm the effects of the drugs, artificial cerebrospinal fluid was microinjected into the DMV in the control group. The results showed no significant difference in gastric motility before and after treatment in this group.

### 3.6. Effect of EA at PC6 on Gastric Motility after Microinjection of L-Glu and GABA into the DMV

Furthermore, we examined the effects of EA at PC6 on rats that received the drug microinjections. L-Glu and GABA were microinjected separately into the DMV. The results showed that EA at PC6 significantly reverse the inhibition of gastric motility by microinjection GABA into DMV (^*∗*^P < 0.05).

### 3.7. Effect of EA at PC6 on Vagus Nerve Discharge via Microinjection GABA in DMV

Based on above data, GABA reverses the efficiency of EA at PC6. Therefore, we determined to verify the output of DMV by EA at PC6. The results showed that microinjection GABA in DMV inhibited vagus nerve discharge, and EA at PC6 promoted vagus nerve discharge (n = 8; Figure 6).

## 4. Discussion

**Figure 3:**
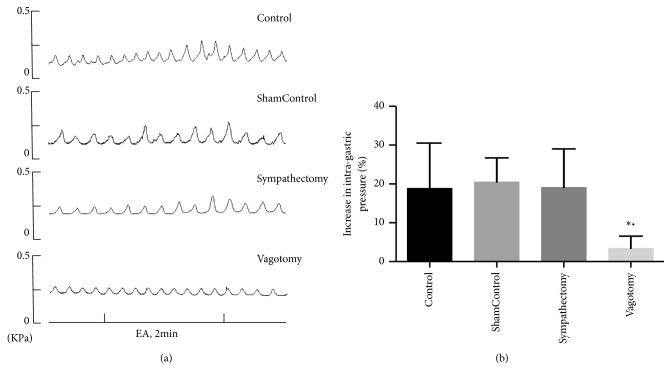
Effect of EA at PC6 on gastric motility via the sympathetic and the parasympathetic pathway. (a) Gastric motility waveforms in the control, sham control, sympathectomy, and vagotomy groups induced by EA at PC6. (b) Percentage increase in gastric motility in the control, sham control, sympathectomy, and vagotomy groups induced by EA at PC6. ^*∗*^P < 0.05, compared with the control group. ^•^P < 0.05, compared with the sham control group (n = 8).

**Figure 4:**
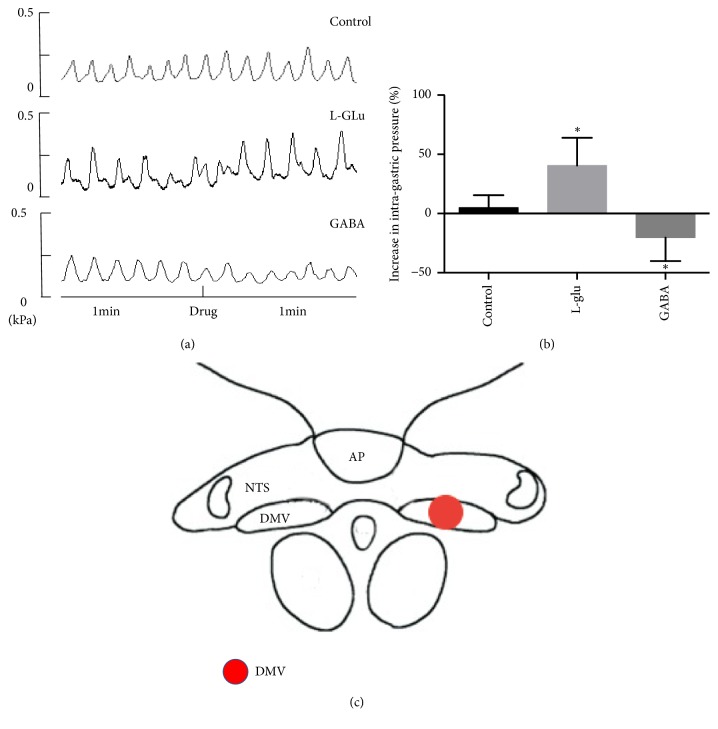
Increase/decrease in gastric motility after the microinjection of different drugs into the dorsal nucleus of the vagus nerve. (a) Wave patterns of gastric motility before and after the injection of artificial cerebrospinal fluid, L-Glu, and GABA into the dorsal nucleus of the vagus nerve. (b) Increase/decrease in gastric motility after the microinjection of artificial cerebrospinal fluid, L-Glu, and GABA into the dorsal nucleus of the vagus nerve. Percentage reduction, ^*∗*^P < 0.05, compared with the artificial cerebrospinal fluid group (n = 8). (c) The location of microinjection to DMV.

EA therapy has been promoted by the World Health Organization (WHO) and National Institutes of Health (NIH) as a surface stimulation therapy [16, 17]. Multiple clinical trials have shown that EA is safe and effective for the treatment of gastric dysfunction [7, 18]. Studies have shown that acupuncture at different sites on the body differently regulates gastric movement; in particular, acupuncture points on the abdomen inhibit stomach movement and acupuncture points on the limbs promote gastric movement [19, 20]. Our findings were consistent with this research. Following EA at PC6 during 30–120 s, the gastric movement was found to be significantly higher than that in the area before needle insertion (Figure 1). Therefore, for the stomach, acupuncture points on the extremities would represent different segments. The stimulation of these acupuncture points led to activation of the parasympathetic nerves and promotion of gastric motility. In contrast, abdominal acupoints represent the same segment, when considering the stomach. When these points are stimulated, the sympathetic nerves become activated and inhibit gastric motility [21–23]. Our research found that the frequency of vagus nerve discharge was significantly higher than that of splanchnic sympathetic nerve discharge following acupuncture at PC6 (Figure 2). After the vagus nerve was cut off, the effect of EA at PC6 on gastric motility had markedly weakened; however, after the splanchnic nerve was cut off, no increased gastric movement was observed following EA at PC6, which suggests that acupuncture at PC6 increased gastric motility by increasing peripheral efferent vagus nerve fiber discharge (Figure 3).

Gastrointestinal movement is mainly dominated by vagus nerve regulation, and brainstem vagovagal neurocircuits are crucial for the regulation of the upper digestive tract [24–26]. Brainstem vagovagal neurocircuits include the DMV, solitary tract nucleus (NTS), and nucleus ambiguus. Therefore, signals are transmitted via vagal sensory afferents from the stomach to the NTS via excitatory neurotransmitters such as glutamate; NTS integrates the signal and input to the superior central nucleus. Another portion of signal transmits to the DMV via GABA and L-Glu and the DMV outputs the signal through the efferent vagus nerve to the stomach. In the signal transduction process of brainstem vagovagal neurocircuits, Glutamate and GABA are the main neurotransmitters. In particular, L-Glu is the major excitatory transmitter in the CNS. Cruz et al. microinjected L-Glu into the DMV of rats and found that the effect at the stomach depends on the specific injection site. The injection of L-Glu into the rostrum of the DMV may lead to excitement of the vagus and increased gastric motility, whereas injection of L-Glu into the posterior of the DMV inhibits gastric motility [27]. A similar study was performed by Zhou et al. who microinjected L-Glu into the DMV and observed the subsequent stimulation of gastric intramuscular cholinergic neurons or nitric oxide (NO)/vasoactive intestinal polypeptide (VIP) neurons that mediated gastric contraction and relaxation [28]. Our findings were consistent with those of other studies. The injection of L-Glu into the DMV in rats increased gastric contraction and motility. Furthermore, GABA is the major inhibitory transmitter in the brain. Studies by Xu et al. showed that nicotinic acetylcholine receptors (nAChRs) activate GABAergic neurons in the tail of the NTS and enhance the inhibition of mouse vagal motor neurons [29]. Previous studies have shown that the microinjection of nonselective ionotropic glutamate receptor antagonist canine uridine into the dorsal vagus complex (i.e., NTS, DMV, and AP) has little effect on gastric motility, whereas microinjection of the GABAA antagonist bicuculline increases gastric motility [30]. Our research showed that the microinjection of GABA into DMV led to a decrease in gastric motility.

**Figure 5:**
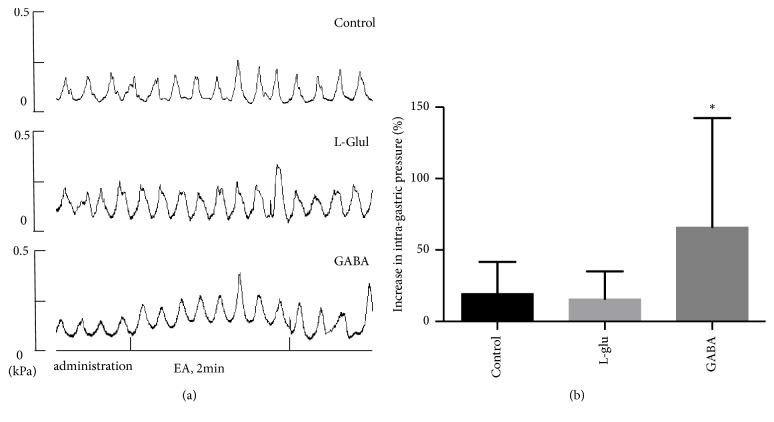
Effect of EA at PC6 on gastric motility after the microinjection of different drugs into the dorsal nucleus of the vagus nerve. (a) Artificial cerebrospinal fluid, L-glutamic acid, and GABA were microinjected to the dorsal nucleus of the vagus nerve, and the wave patterns of gastric motility during EA at PC6 were recorded. (b) Increase in intragastric pressure induced by EA at PC6 after the microinjection of artificial cerebrospinal fluid, L-Glu, and GABA into the dorsal nucleus of the vagus nerve. ^*∗*^P < 0.05, compared with the artificial cerebrospinal fluid group (n = 8).

**Figure 6:**
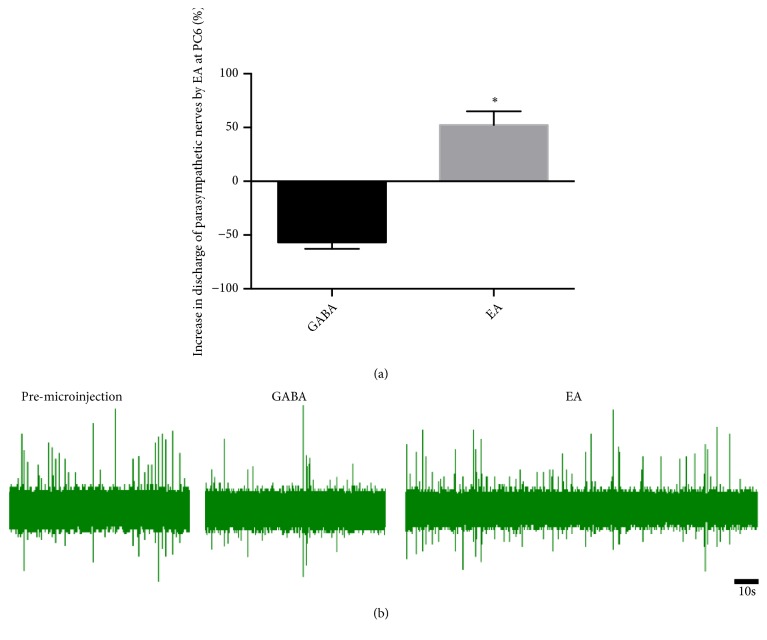
Effect of EA at PC6 on vagus nerve discharge via microinjection GABA in DMV. (a) Percentage increase of parasympathetic nerve discharge during microinjection GABA and EA. ^*∗*^P < 0.05, compared with the microinjection GABA (n = 6). (b) Parasympathetic waveform of microinjection and EA at PC6.

To investigate the effect of EA at PC6 in the brainstem vagovagal neurocircuits, the rats in the present study that received L-Glu and GABA microinjections into the DMV also received EA at PC6. As observed in Figure 5, the effect of EA at PC6 on gastric motility did not differ significantly between the L-Glu and control groups. However, the gastric motility was significantly increased in the GABA group as compared to that in the control group. Moreover, our research also provides microinjection of GABA into DMV also inhibiting vagus nerve activity, and EA at PC6 reverses it (Figure 6). Therefore, EA at PC6 can reverse the GABA signals which transmit into DMV, thus reducing the inhibitory effect on the vagal efferent motor neurons.

In conclusion, EA at PC6 reverse the inhibition of efferent vagal motor fibers to the DMV—mainly through the inhibition of GABA transmission—and thus promotes the activity of efferent vagus nerves and increases gastric motility.

## Data Availability

The Excel data used to support the findings of this study are included within the Supplementary Materials files (available here). No additional unpublished data are available.

## Abbreviations

EA: Electroacupuncture
DMV: Dorsal motor nucleus of the vagus
NTS: Nucleus tractus solitarii
GABA: Gamma-aminobutyric acid
L-glu: L-glutamic acid
NO: Nitric oxide
VIP: Vasoactive intestinal polypeptide.

## Additional Points

Mechanisms of EA in adjusting gastric motility via brainstem vagovagal neurocircuits remain largely unknown. The aim of this study was to investigate mechanisms of EA in adjusting gastric motility. EA at PC6 improves gastric motility via brainstem vagovagal neurocircuits and GABA transmission. This study further proves the effects and mechanisms of EA for adjusting gastric motility. It will help complete the biological mechanism of EA.

## Supplementary Materials

Supplementary Materials Sheet1 (gastric pressure) was used to prove the effect of EA at PC6 on gastric motility (control group, vagotomy group, and sympathectomy group). Sheet2 (nerve discharge) was used to prove the effect of EA at PC6 on autonomic nerve discharge. Sheet3 (microinjection) was used to prove the effect of L-Glu and GABA microinjection into DMV on gastric motility and the effect of EA at PC6 on gastric motility after microinjection of L-Glu and GABA into the DMV.
